# A survey-based design of a pricing system for psychotherapy

**DOI:** 10.1186/s13561-018-0213-7

**Published:** 2018-11-13

**Authors:** Beat Hulliger, Martin Sterchi

**Affiliations:** Institute for Competitiveness and Communication, University of Applied Sciences and Arts Northwestern Switzerland (FHNW), Riggenbachstrasse 16, 4600 Olten, Switzerland

**Keywords:** Survey, Health insurance, Health care pricing, Tariff system, Regulation, C83, I13, I18

## Abstract

For admission to statutory health insurance, it is common in Switzerland that health care providers negotiate prices for health care services directly with health insurers. Once they agree upon a price, they must submit the resulting price to the Federal Office of Public Health (FOPH), which can then authorize it. Swiss law requires the prices in health care to be based on empirical data. There has been little research on how to derive such a price for health care from empirical data and which data should be used. Based on a collaboration with psychological psychotherapists in Switzerland, we have designed a pricing system. The empirical basis were two representative surveys: a survey about costs and earnings of psychotherapists, as well as a time-use survey for psychotherapy. This paper shows the methodology followed to establish an empirically based pricing system. The paper may serve as a practical guide for health service providers who want to develop a pricing system. Our approach offers a high degree of freedom because it involves the collection of the data and an explicit modelling phase. At the same time, it might be more resource intensive than other approaches that are based on existing data sources.

## Introduction

The Swiss health care system comprises four main stakeholders. First, there are the resident citizens who are required to have statutory health insurance by law. Second, there are health care providers such as hospitals, medical practitioners and others. Third, private competing insurance companies provide statutory health insurance, as well as supplementary health insurance. The fourth important stakeholder, the government, regulates statutory health insurance. On a national level, most tasks with respect to health care are assumed by the Federal Office of Public Health (FOPH). FOPH authorizes the insurance premiums and oversees the scope of mandatory coverage of health services, among other things. In addition, the 26 cantons have a critical role since they are the main political entities responsible for health care in Switzerland. The cantons license insurance providers, organize the health care offered in hospitals and manage the subsidies for health care institutions, among other things [1].

In Switzerland, a fee-for-service scale called TARMED regulates the price of outpatient health care by medical doctors. It categorizes all the services of outpatient health care and contains a relative cost weight for each of these services. Based on this relative cost weight, the health insurance companies and health care providers negotiate the effective price for a service in every canton and on a yearly basis [1]. However, TARMED only applies to care providers with a medical degree. In particular, it regulates psychotherapeutic services offered by medical doctors with a psychiatric specialization. In recent years, the national government expanded the scope of statutory health insurance to include more non-medical care providers. The most recent expansion concerned the neuropsychologists. The national government officially recognized them as care providers in December 2016 [2]. This allows them to provide care independently and at their own account within the framework of statutory health insurance.

In 2012, the Federal Law on Psychological Professions (PsyG) became effective [3]. It defines the requirements for psychotherapists to obtain the psychotherapy practice license and in particular, it sets educational standards for psychological psychotherapists. This law serves as the basis for the extension of statutory health insurance to cover psychological psychotherapists. The Swiss Federal Health Insurance Act (KVG, Art. 43) defines that health care providers should negotiate the prices directly with the health insurers. The Swiss Federal Council only interferes if the negotiations do not succeed. In collaboration with three associations representing the psychological psychotherapists, a pricing system for psychotherapy, which will be the basis for the negotiations with the health insurers, was developed. During all stages of the process of designing the pricing system, a close collaboration of the research team with a project group that consisted of representatives from the three associations of psychotherapists, among them practicing psychotherapists, ensured proper alignment with the objectives of the project.

For a pricing system two elements are required: i) the tariff structure, which is a systematic nomenclature with the exact definitions of the services of psychotherapy including the units of measurement and ii) a relative price scale for each position of the tariff structure. The price scale is expressed in terms of tax points and is set in such a way that one tax point corresponds to approximately one Swiss Franc. The challenges for establishing a pricing system are manifold. First, the services must be mapped correctly and coherently onto the tariff structure. Second, the tariff structure must be sufficiently precise and detailed such that psychotherapists can clearly and unambiguously assign their time spent to the appropriate position in the tariff structure. Likewise, the tariff structure must be sufficiently general in order to fulfil the requirements of all types of psychotherapists (e.g. general psychotherapy vs. emergency psychotherapy). Nevertheless, the tariff structure should be simple enough such that it can be applied in practice without too much effort. Finally, yet importantly, the pricing system must allow an efficient practitioner to achieve a fair income. In other words, a psychotherapist satisfying some predetermined share of therapy-related (billable) activities must be able to cover all their costs and earn a fair income. However, the question of what a fair income for a psychotherapist is will be the subject of negotiations between the stakeholders.

Apart from a vast body of literature about systems based on diagnosis related groups (DRG) (e.g. [4]), there is little published literature on designing pricing systems for statutory health care. However, the Swiss regulatory body outlines some principles for designing such a pricing system. First and foremost, statutory health insurance must be non-profit [1]. Accordingly, the Health Insurance Ordinance (KVV, Art. 59c, 1b) states that the price can only cover the costs necessary for an efficient service provision. Furthermore, it defines that the underlying costs need to be disclosed (KVV, Art. 59c, 1a). In other words, the pricing system needs to be transparent and it must be based in some way on empirical data. However, it does not define how the empirical data should be collected and how it should be used in designing a pricing system.

In this paper, we propose a pricing system for psychotherapy that relies upon the results of a representative survey about the costs and earnings of psychotherapists, as well as a representative time-use survey. This paper may serve as a pragmatic guide for other health care providers attempting to establish a pricing system in health care. We present the methodological choices faced and the rationale for decisions made. Due to confidentiality agreements with the three associations representing the psychotherapists, we cannot reveal any specific results of our approach. Nevertheless, the description of the approach should help to stimulate the discussion about the methods of establishing a tariff system.

This paper is organized as follows. Section Methods explains how the tariff structure was developed and describes the design, execution, data preparation and analysis of the costs and earnings survey and of the time-use survey. Section Results presents the results and explains how we used the empirical results to establish a pricing model for the computation of the final price for psychotherapy services. Section Discussion discusses some problems and possible extensions of our approach. Finally, Section Conclusion concludes.

## Methods

### Tariff structure

Before a price can be established, the goods to be priced must be clearly defined. In the case of psychotherapy, the goods are services. The systematic nomenclature of psychotherapy services is the tariff structure. It represents the different health care services offered by psychotherapists in a structured way such that they can be aggregated into categories of services. For every service, the mode of delivering the service is determined. A psychotherapy session, for example, can be held as a face-to-face meeting, but also by phone or even online. In addition, a psychotherapy session involving a group or the parents of the patient is different from a one-on-one session, first because the work of the psychotherapist is different and secondly because the amount charged for the psychotherapy session may be split up among the patients of the group session. Furthermore, for some services such as the evaluation of psychological test results there is no physical presence of the patient necessary. The tariff structure precisely describes every service and defines the billable unit. A psychotherapy service is either priced overall, or, as in other service-oriented tariffs, the service is priced per time used. The elements in the tariff structure are based on 5 min-units, i.e. the smallest billable unit are 5 min of a service. However, some services such as writing a formalized report for the health insurers are priced overall and are thus reimbursed with a flat rate price.

The project team developed a first version of a tariff structure in several rounds of discussion. Qualitative interviews with several psychological psychotherapists helped to structure the tariff. For the further development of the tariff structure, a workshop with psychotherapists was organised in order to establish a better understanding of the domain and of the different services of psychotherapy. The participating psychotherapists were from different fields such as general psychotherapy, psychotherapy for children and adolescents, psychotherapy for elderly people, and emergency psychotherapy. The goal of the workshop was to find out, which services need to be taken into account and whether or not the proposed tariff structure was covering the needs of various types of psychotherapy in a useful way. In order to have a concentrated discussion, four fictional cases of psychotherapy were submitted to the participants. The cases were meant to capture many facets of psychotherapy and were sent to the participants prior to the workshop. The fictional cases also covered typical situations such as a patient not showing up for a meeting, an emergency meeting or a psychotherapy session in special conditions such as accompanying patients when using public transport. After the integration of the outcomes of the workshop, a next version of the tariff structure was developed. This version was used in a pilot study with ten volunteering psychotherapists. The participants reported all their activities during one week as if the tariff structure was already in place. The tariff structure was further adapted by taking the outcome of the pilot study into account. Thus, the tariff structure evolved in five feedback loops. This included adding and removing positions or simply clarifying certain positions. The elements of the tariff structure served as the basis for the time-use survey.

### Survey about costs and earnings

A major issue for the survey on costs and earnings is that psychotherapists in Switzerland work in different economic models [5]. While many psychotherapists work independently and at their own account, there are psychotherapists who are working on behalf of a psychiatrist or a general practitioner (GP). In that case, the medical doctor (psychiatrist or GP) is the responsible therapist and thus the treatment is eligible for coverage through statutory health insurance under TARMED. This model of psychotherapy is called delegated psychotherapy. Furthermore, there are psychotherapists who are employed by an in- or outpatient facility for mental health care, which bills psychotherapy costs under a special tariff. Finally, a considerable number of psychotherapists exhibit some combination of the aforementioned models and assume further activities such as academic teaching. As a result, most of the psychotherapists’ independent work is part-time. Hence, the survey needed to account for those different economic models. For example, a psychotherapist who works independently and at their own account while, at the same time, is also employed by a psychiatrist needs to be able to capture their costs and earnings for the different models separately. Another complication is that some psychotherapists work in multiple practices and keep separate accounts.

A pilot survey clearly showed that the respondents cannot be asked for detailed accounts of all these activities separately. The questionnaire, although implemented in an online tool, was too complex. Therefore, we redesigned the questionnaire in order to define the most important independent primary practice with respect to costs and earnings. In other words, a participant working in more than one practice must only report the costs and earnings of the practice in which they worked the most during the survey period. Finally, several psychotherapists may work together in a group practice and thus share the costs. In that case, participants were asked to only report their share of the costs.

The survey focused on the costs and earnings of psychotherapists in 2014. Of particular interest are the costs and earnings of the psychotherapists who work independently and at their own account since this economic model resembles the future of psychotherapy the most. Nevertheless, the survey also captured the costs and earnings of delegated psychotherapists. This procedure has two advantages. First, it allows us to compare the independently working psychotherapists with the delegated psychotherapists. Secondly, it triggered participants working in both models to separate costs and earnings of the different models and therefore enforced consistent answers. However, the inclusion of delegated psychotherapists involved a delicate filtering scheme at the beginning of the questionnaire. The filtering prioritized the independent economic model as long as the psychotherapist worked at least 8 h per week in this model. Psychotherapists working less than 8 h per week as independent psychotherapists were still questioned about costs and earnings as delegated psychotherapists as long as the workload in this model accounted for at least 8 h per week. For all other psychotherapists, no questions about costs and earnings were asked.

Next, the questionnaire contained some general questions about the working model, including forms of collaboration, facilities, number of employees and subcontractors. We also asked participants if their practice was set up in 2014 or if it was shut down during the course of 2014 in order to determine non-representative observations.

As for the costs, we asked participants to report their acquisitions as well as their operational costs. The positions are listed in Table 1. Certain positions were further divided; for example, salaries were broken down into net income, social security contributions, pension fund contributions and insurance premiums. Positions that were unclear were explained with examples. Moreover, participants had the option to specify further costs manually in the form of free text input. In order to check the consistency and plausibility of the reported costs, we asked participants to indicate their earnings as well. Earnings were subdivided based on who paid for the treatment. Hence, the main categories were private patients/supplementary health insurance, accident insurance and disability insurance, among others.

**Table 1:** Overview of cost positions that participants of the survey had to answer

Acquisitions	
Furnishing	
Electrical equipment	
Telecommunication devices (including computers)	
Vehicles	
Therapy-related acquisitions	
Psychological test material and other acquisitions	
Operational costs	
Lease costs	
Transportation expenses	
Staff costs	
Salaries	
Training costs	
Interest	
Telecommunication	
Office supplies	
Insurance fees	
Marketing and accounting expenditures	
Therapy-related costs (books, tests, etc.)	

In addition to costs and earnings, the survey asked participants to specify the average workload in the primary practice in hours per week and work weeks per year. This information was necessary to compute the average level of capacity utilization for every participant. This was crucial since most of the participants work part-time as psychotherapists and hence, in most cases, the reported costs and earnings correspond to a part-time workload. For example, a participant working only one day as an independent psychotherapist was supposed to indicate only the costs related to this workload. Consequently, the responses were standardized in the processing phase (see Section Processing of survey results) in order to represent the costs of a full time employment.

The population of interest are all psychological psychotherapists in Switzerland with a federal license. Since the register of FOPH containing all relevant psychotherapists was not operational at the time of the survey, the address databases of the three associations of psychological psychotherapists involved in the project were used as sampling frame. The information about the practice license was used to delimit the members of the associations who should participate in the survey. However, there is a number of psychotherapists who are federally licensed but are not a member of an association [5]. The population coverage by the members of the associations was estimated to be at least 87%. Hence, the sampling frame was restricted to the members of the associations with a psychotherapy license. The sampling frame contained *N* = 4297 psychotherapists (Table 2). Since a considerable non-response must be expected in such a time consuming survey, the sample was exhaustive, i.e. the full population was surveyed. This resulted in a sufficiently large net sample size of 1336 observations before data processing, whereof 466 observations correspond to psychotherapists who work, at least partially, as independent psychotherapists.

**Table 2:** Size of sampling frame and number of completed questionnaires

Sampling frame (N)	4297
Completed questionnaires (total)	1336
Independent psychotherapists	466
After processing	355

The survey was administered with the software *Questback* that allowed participants to enter their data online. The questionnaire was available in German and French. The survey allowed participants to report their answers within a period of 3 weeks. They received an information letter and an invitation by e-mail and they were able to contact the survey team via a generic e-mail address. After 2 weeks, a reminder with full support of the psychotherapy associations was sent out and the fieldwork was extended by 2 weeks to allow more participants to finish the data entry. At the end of the questionnaire about the costs and earnings, survey participants were asked to choose one out of three subsequent weeks to participate in the time-use survey. The participants were advised to choose a week that is representative of their typical workload.

### Time-use survey

The design of a time-use survey involves a substantial amount of methodological considerations about the mode of the survey, the coding scheme and follow-up probes [6]. The mode of the time-use survey was similar to that used for the survey about the costs and earnings. Hence, the time-use survey was conducted online with the software *Questback*. The advantage of the online mode compared to more traditional paper and pencil approaches or approaches involving *Microsoft Excel* was more control at the input stage, more coherent reporting and more control for the survey managers about the response behavior. The participants reported their activities on a daily basis during a week including the weekend. One reason why weekends were included was because some psychotherapists are part of an emergency service and thus, they might report activities on weekends as well. Furthermore, it is common for psychotherapists to see patients on Saturdays. We notified participants by e-mail before the start of the week they had chosen at the end of the costs and earnings survey. Every morning during the survey period, an e-mail with the link to the survey was sent to the participants in order to motivate them to report their activities promptly.

In order to conduct a clear and meaningful time-use survey, we needed to provide a comprehensive list of possible activities of psychotherapists, i.e. a coding scheme, from which the participants could choose. Generally, the list of activities reflected the elements of the tariff structure. However, it was necessary to add further elements in order to cover the range of possible activities completely. For example, we included activities such as work breaks, the waiting time in-between patients and administration and organization of the practice. Finally, with ‘*other activities’* we were able to capture any other activities that could not be assigned to an activity of the list. Overall, participants could choose from a list of 25 activities. We asked participants to report their activities chronologically and provided 40 possible entries per day. However, reported activities and their order could be revised at any time. To avoid typing errors participants could select their answers from a drop-down menu. In addition to the activity coding, the time spent on every activity was required. The time unit was 5 min as for the tariff. Again, participants were able to choose from a drop-down menu of time periods starting with 5 min and ending at 600 min. Furthermore, we asked participants to indicate the mode of work, the mode of communication and whether the activity reported corresponded to an emergency or not. For the mode of work, participants could choose between independent work, work resulting from an employment by a psychiatrist, i.e. delegated psychotherapy, or activities in a mental health care facility. The possible modes of communication were face-to-face, by phone or online.

In addition to the activities, we asked participants to specify the start and end time of the respective workday, which allowed us to check whether the reported activities were consistent with the total daily work time or not.

Of the 551 psychotherapists who agreed to participate in the time-use survey, 321 completed the questionnaire.

### Processing of survey results

Survey data typically contains missing values and outliers and is inconsistent in many different ways. Therefore, the data must be processed before starting with the analysis. In the paragraphs that follow, the performed processing of the costs and earnings data is explained in detail. At the end of this section, we briefly describe the processing of the results of the time-use survey.

Implausible observations in the survey about the cost and earnings were discarded. One source to assess the plausibility of an observation was the participant’s comments. For example, observations were dropped if the participant specified that the indicated costs and earnings were not representative for their usual work situation. Furthermore, observations were discarded if either all costs or all operational costs were missing. Observations with missing earnings were not discarded although in those cases the cross-checks between costs and earnings were not possible. Discarding observations due to non-reporting of earnings would have reduced the sample size dramatically. It seems that a majority of the participants experienced difficulties indicating their proper earnings.

Based on common accounting principles, we transformed reported values for acquisitions into amortization values, which represent the yearly cost of an acquisition. The current linear depreciation rates of the Swiss tax authorities for business entities were used as amortization rates [7]. For example, if a psychotherapist acquires a computer for 3000 Swiss francs, the corresponding amortization rate is 20%.^1^ Hence, the psychotherapist needs to take into account yearly costs of 600 Swiss francs for the acquisition of the computer. Critics might argue that the survey should ask participants to report amortization values instead of acquisition values. However, in order not to increase the complexity of the survey any further, only the acquisition values were collected.

Imputation of missing lease costs and salaries was necessary because of the importance of these two positions. They turned out to be the most substantial cost drivers of independent psychotherapy. As for the lease costs, the survey contained different questions depending on whether a psychotherapist owns the rooms for the practice or only rents them. In the latter case, the participants were asked to indicate the lease costs (on a yearly basis) as well as the supplementary costs such as costs for heating and electricity. However, if the participants own the rooms for their practice, they were asked to declare the imputed rental value, a concept used in the computation of taxes in Switzerland. The supplementary costs were added either to the lease costs or to the imputed rental value in order to get the gross rental costs per year. If the resulting gross rental cost was less than 2000 Swiss francs, we assumed that the participant erroneously indicated the monthly cost instead of the yearly cost. In those cases, we multiplied the amount by 12 in order to impute yearly costs. With regard to the salaries, a number of participants did not indicate their salary but all their other costs and earnings. In that case, the difference between earnings and costs was imputed as a proxy of their salary. Furthermore, some participants indicated zero costs for positions that, by definition, cannot be zero. For example, every psychotherapist who works independently must exhibit costs arising from social security contributions. Therefore, such zero values were set to missing values in order not to distort the statistical computations (Section Statistical analysis) with zeros that are in fact missing values.

Finally, as discussed above, comparability of costs requires standardizing all the observations related to a part-time workload. For this purpose, the capacity utilization level was determined for every participant. Based on the average workload in the primary practice expressed in hours per week and weeks per year, the average workload per year in hours could be determined for every participant in the sample. Following the recommendations of the professional associations [8], observations with at least 1824 h per year were considered a full time workload. The workload of observations with less than 1824 h per year was determined proportionally. A psychotherapist working, for example, 900 h per year exhibits a workload of 49%. The capacity utilization level was used to standardize all costs except for the amortization values of the acquisitions and the gross rental costs. The rationale for excluding the amortization values from the standardization process is that at least part of these costs are fixed, in other words, they do not vary with the capacity utilization level. For example, psychotherapists acquire office furniture regardless of whether they work one or five days a week. Moreover, a comparison of the gross rental costs with the capacity utilization levels showed that for some observations rental costs are disproportionately high. This implies that in some cases, the capacity of a practice might not be used efficiently. Therefore, the amortization values of the acquisitions and the gross rental costs were standardised using factors that are smaller than the corresponding capacity utilization levels. This ensured that acquisition costs and gross rental costs were not overstated. As can be seen in Table 2, the final sample after processing contained 355 observations.

As for the time-use survey, we first imputed the time for mandatory work breaks based on Swiss law since work breaks were in many cases not reported properly. Hence, for every 4 h of work, we imputed a break of 15 min. Based on those imputed values and all the other activities reported by the participants, we were able to compute the total workload for each participating psychotherapist. We then discarded observations with a workload of less than 8 h per week because we considered such a workload as not representative. The final sample contained 187 observations. Finally, we aggregated the time for every activity over all participants and divided the sum per activity by the overall number of hours worked, thus arriving at a weighted proportion where the weight is proportional to the total work time per participant. As a result, we found a percentage for every activity that is likely to represent a psychotherapist’s typical workday. As a hypothetical example, we might have found that a psychotherapist typically spends 10% of their time on the administration and organization of their practice, 70% on psychotherapy sessions, and so forth.

We carried out the processing of the data, as well as the statistical computations (Section Statistical analysis) in R [9].

### Statistical analysis

In order to use the survey results for designing the pricing system, we needed to calculate average costs and earnings for every position that are a good representation of a typical psychotherapy practice. An obvious choice for summarizing data is the arithmetic mean. However, using the arithmetic mean in our case would have serious drawbacks due to the characteristics of the data. First of all, the data contains outliers that tend to inflate the arithmetic mean. This is especially problematic if outliers are the result of measurement error, i.e. misreported costs or earnings. Secondly, the data contains many observations that are zero and thus exhibit characteristics of a semi-continuous distribution with a peak at zero. Furthermore, we decided that observations should be weighted according to the capacity utilization level. In other words, we consider participants with a high capacity utilization level as more representative for a typical psychotherapist’s practice than those with a low capacity utilization level. For all those reasons, we decided to summarize the data by using a trimmed, weighted mean that accounts for zero-inflated variables with the weights being the capacity utilization level.

Our concerns about selection bias were mitigated as the responding sample corresponded well with the population shares of the available covariates. Thus, no additional poststratification weights were applied. Potential selection bias may arise as the result of two scenarios. First, psychotherapists with a low income may have higher non-response rates than the rest of the psychotherapists due to stress. Secondly, psychotherapists who desire to change their working conditions may have lower non-response rates than the rest of the psychotherapists. Both scenarios can lead to a bias in average costs. We could imagine similar effects if highly efficient psychotherapists exhibit a low non-response, and/or inefficient psychotherapists exhibit a high non-response. The sample structure of respondents gives no clear indication of bias in one direction or the other. The discussion of the survey results with practitioners and the project team has shown that our results are credible.

Trimming of extreme observations is a commonly used robustification method. It relies on the assumption that the bulk of the data has a normal or near-normal distribution and that a few outliers occur on both sides of the main distribution. If the data has a semi-continuous distribution with one discrete mass (in our case the zero observations), then this assumption does not hold anymore. One way to treat outliers is to separate the discrete part before trimming is applied and add the discrete value with its appropriate weight after trimming. In addition, we often need to compute a weighted estimator. A detailed description of our estimation procedure is provided in the appendix. In the section that follows, we will show how the statistical results of the costs and earnings survey were combined with the results from the time-use survey in order to compute the price of psychotherapeutic care.

## Results

**Fig. 1:**
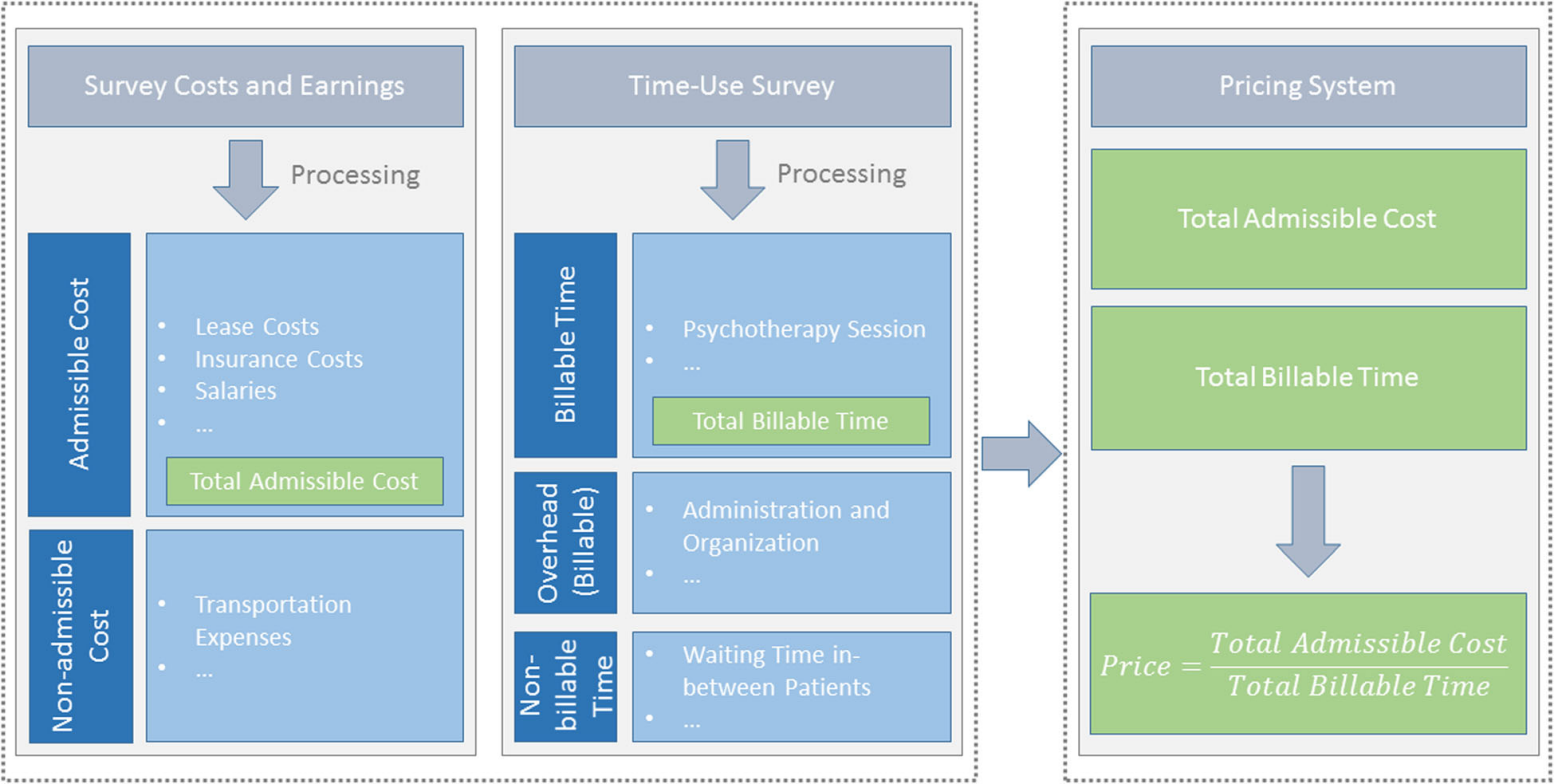
Schematic representation of the elements in the pricing model

The key elements for the computation of the price are the results from the survey about the costs and earnings and the time-use survey. However, the survey results represent the current situation of psychotherapists whereas the price must be based on costs and a time-use that represent the future work situation of psychotherapists under the new tariff system. Hence, it was crucial to transform and adjust the survey results into a pricing system that models the future situation of psychotherapists appropriately. This included: i) omitting certain costs reported in the survey, ii) adding costs not contained in the survey and iii) deviating from the survey results in some cases. Similar to the time-use survey, the primary task was to define which activities are covered under statutory health insurance. A more detailed account of the steps necessary is given in the following paragraphs and Fig. 1 illustrates the model.

First of all, a careful analysis of every cost position was conducted in order to determine the total cost that is admissible under statutory health insurance. For every cost position, we defined whether it should be included in the computation or not. The transportation expenses illustrate this point. They were included in the survey in order to have a comprehensive set of costs but were not considered in the final pricing model. Including the transportation expenses in the computation of the price for psychotherapy services would be hard to justify in negotiations since having a car, for example, is not necessary to practice psychotherapy and the costs thereof should not be billed to patients. In a second step, certain cost positions, which were not considered in the survey, were added. For example, under a statutory health insurance scheme a professional billing software is required, which the survey did not account for. Hence, based on the standards applied by medical practitioners we added yearly costs for a billing system. Finally, some of the survey results were implausible or not applicable for other reasons and, therefore, we decided to deviate from the survey results. Most importantly, we did not consider the net income as expressed in the survey results. As mentioned above, the pricing system should represent the future work situation of psychotherapists in Switzerland. Therefore, the pricing system was set up such that the net income could be entered manually as an external parameter. The benefit of this approach is that the pricing system is sufficiently flexible, especially if we consider that the net income may be the most controversial cost element discussed in negotiations between the health care providers and health insurers due to its relative importance. Another example where we were deviating from the survey results are the pension fund contributions. The resulting average value of pension fund contributions in the survey was considered too low compared with professional standards in Switzerland. Therefore, based on the net income and the prevailing contribution rates in Switzerland, the pension fund contributions were recalculated. It is important to note that for all deviations from the survey results a clear argumentation and well-documented external sources were provided to the project partners. After adding, removing and modifying some cost positions, the remaining costs were added to yield the total yearly cost that is needed for an efficient full time psychotherapist and thus should be covered by statutory health insurance.

Secondly, we determined for every activity in the time-use survey whether it is relevant for statutory health insurance or not. For example, the health insurers do not cover the waiting time in-between patients. Thus, the percentage share of waiting time in-between patients is not relevant for statutory health insurance. In addition, for all activities that are relevant for statutory health insurance, we specified whether the activity is directly billable to patients or forms part of the overhead. For example, the administration and organization of the practice is obviously a necessary task in any medical practice and should be covered by the practice’s income. However, it is not directly billable to patients and hence, the activity is part of the overhead and its costs are spread proportionately over all the billable activities. Furthermore, activities such as the mandatory work breaks as well as activities that are compensated by a flat rate price are excluded from the billable time. The result of the steps mentioned in this paragraph is a list of necessary activities for psychotherapy that are either directly or indirectly billable. The time shares of these necessary activities are restandardized to add up to 1. This last step is crucial because we want to pass on the total cost of a full time psychotherapist to the billable part of a full time workload. Restandardizing the time shares makes sure that we do not underestimate the billable part of the workload. One final important step is the definition of the disposable weekly work time. In Switzerland, it is common to work for 42 h per week. However, since mandatory work breaks are not part of the list of directly or indirectly billable activities, the time for the mandatory work breaks is subtracted from the 42 h. Furthermore, a weekly constant for further education, which is compulsory by law (PsyG, Art. 27, b) is also subtracted from the weekly normal work time. This reduces the average disposable weekly work time to 37.7 h.

Finally, we had to set the costs and the work time on equal footing. The costs admissible under statutory health insurance were calculated on a yearly basis. Thus, they must be divided by the number of weeks worked per year (43 weeks^2^) in order to compare them with the weekly work time. Then, weekly costs admissible under statutory health insurance are divided by the billable time share of the disposable weekly work time. The result is the cost per minute. As was mentioned above, psychotherapy services are billed based on 5-min units. Hence, the cost per minute multiplied by 5 is the price for any type of psychotherapeutic activity that can be billed. The computation of the price can be summarized with the following formula:

$$ Price=\frac{Total\ admissible\ cost\ \left( Swiss\ francs\right)}{Total\ billable\ work\ time\  per\  week\ \left(\mathit{\operatorname{Min}}.\right)}\times 5 $$

As in TARMED, the price for all services is the same. To give an example, assume that weekly costs admissible under statutory health insurance are 5000 Swiss francs and further assume that the time share of billable activities is 80% which corresponds to 1810 min per week. In that case, it results a price of 13.81 Swiss francs per 5 min. It is crucial that the costs admissible under statutory health insurance are only divided by the time share of billable activities. This way we implicitly pass on overhead activities such as the administration and organization to the price.

A complete pricing system for psychotherapy, or more generally for health care, requires additional specifications. Firstly, the flat rate prices for services such as writing formalized reports for health insurance companies need to be specified. Secondly, some mark-up over the regular price is needed for emergencies, which is common in pricing schemes in health care. Finally, the travel time of psychotherapists to visit patients should be compensated. This last point is particularly important since, in our approach, transportation expenses are not included in the admissible cost. Most of these additional specifications can be based on existing regulations in TARMED with some adjustments for the special case of psychotherapy.

The calculations for the pricing model are implemented in a *Microsoft Excel* worksheet such that the parameters can be entered manually (e.g. net income) and, at the same time, the basic input from the surveys remains separated from the pricing model.

## Discussion

The results of this study show that it is possible to build a pricing system for psychotherapy based on the results of a survey about the costs and earnings and a time-use survey. However, as we have seen above, the design of the pricing system involves methodological decisions on many different levels. As there exists relatively little research in this field, many of those decisions were based on the domain knowledge of the project group that actively followed the progress of this study. In many other cases, we were able to base our decisions on principles that are already implemented in the Swiss health care system. For example, we decided that further education should not have an effect on the price a psychotherapist can charge. The reason for that is to conform to TARMED. It specifies a unique price for every service in outpatient health care regardless of individual attributes of the medical practitioner performing the service such as further education or experience.

Moreover, our approach might suffer from limitations that are commonly known in health care systems. One such problem might be moral hazard [10]. Psychotherapists might have an incentive to advise patients to seek more hours of therapy than is necessary. Currently, Swiss health care authorities address this problem as follows: a psychiatrist is free to mandate 40 sessions of therapy that are covered by statutory health insurance. If more therapy sessions are necessary, the psychiatrist must write a report for the attention of the health insurance company of the patient. The latter then decides whether it covers more than 40 sessions of therapy or not. It is therefore likely that such a rule will also apply to psychotherapists once they are admitted to statutory health insurance. In addition, health insurers supervise and monitor the efficiency and expenditures of health care providers. Therefore, a psychotherapist who would have an extraordinary cost or therapy structure would have to justify this and, ultimately, could be excluded from the health insurance system.

Another limitation of our approach might be the lack of considering treatment quality. A pricing system where the price of a treatment depends on its quality can be beneficial for patients as it may incentivize psychotherapists to improve the quality of their care. However, there are certain problems with treatment quality. First of all, one of the premises of the Swiss health care system is that medical practitioners do not have to guarantee the success of a treatment. In other words, medical practitioners are reimbursed regardless of whether the treatment was successful or not as long as care was provided in all conscience. Secondly, the measurement of treatment quality is in itself a challenging endeavor and there exist different approaches [11]. One approach, for example, is based on assessing the outcome of a treatment. While the measuring of outcomes is already hard for many physical diseases, it may become impossible for the various forms of mental disease. Although treatment quality is difficult to determine, psychotherapists are usually involved in supervision processes where either they supervise the work of a colleague or they receive advice from a colleague about their own cases. Furthermore, as mentioned above, minimal further education is required by law. Hence, two mechanisms already in place have the aim of ensuring quality of treatment.

## Conclusion

The purpose of this paper was to outline the process of designing a pricing system in health care. The different steps are explained through the example of psychological psychotherapy in Switzerland. According to Swiss regulation, the proposal of a pricing system needs to be based on a transparent empirical data basis. Hence, this paper set out to combine the results of two surveys in order to design a pricing system. A survey about the costs and earnings of psychotherapists helped to determine the essential costs incurred when practicing psychotherapy, while a time-use survey served as the basis for learning what share of the work time is directly billable. Dividing the relevant total cost by the billable time resulted in the price for psychotherapy. Together with the tariff structure, this price builds the core of the pricing system.

Overall, this paper shows that it is possible to design a pricing system for health care based on survey results. However, the design involves many methodological decisions that often require a sound knowledge of the concrete domain studied. This can be achieved by conducting, for example, workshops with the practitioners or pilot studies. Moreover, the pricing system crucially relies on the validity of the survey results. Hence, a comprehensive and sound design of the surveys is critical for the whole project. In addition, the pricing system is always just a model of the reality and certain factors such as false incentives or quality of treatment cannot be directly built into the pricing system but must be addressed in a more general way accounting for common principles of the whole health care system.

As was previously mentioned, it is not possible to present numeric results due to confidentiality agreements. This is a major limitation of this paper. Future research should assess the procedure that is proposed in this paper and hopefully can present numeric results.

## Appendix

Let the data be *x*_*i*_ (*i* = 1, …, *n*) with weights *w*_*i*_ (*i* = 1, …, *n*), denote the sample by *S* and let *Z* denote the set of the indices of the observations with a value of 0, i.e. *Z* = {*i*| *x*_*i*_ = 0}. Estimate the proportion of the observations with a value of 0 in the population with *p*_*z*_ = ∑_*i* ∈ *Z*_*w*_*i*_/∑_*i* ∈ *S*_*w*_*i*_. To simplify the treatment assume that ∑_*i* ∈ *S*_*w*_*i*_ = *n*. Let the trimmed mean of the non-zero observations be *m*_*tnz*_. Note that for the trimmed mean with weighted data the weighted quantiles must be calculated and the weights for *i* ∈ *Z* must be set to 0 before calculating *m*_*tnz*_, the trimmed weighted mean of the non-zero observations. Assuming *x*_*l*_ and *x*_*h*_ denote the lower and upper trimming quantiles, then *n*_*t*_ = ∑_*S* ∖ *Z*_*w*_*i*_ 1{*x*_*l*_ ≤ *x*_*i*_ ≤ *x*_*h*_} denotes the number of non-trimmed observations (or, more generally, the sum of the weights of the non-trimmed observations).

The estimator for the population mean is

$$ {m}_t={p}_z\cdotp 0+\left(1-{p}_z\right)\cdotp {m}_{tnz}. $$

The variance estimator must take into account the variability of *p*_*z*_ and *m*_*tnz*_. Under the assumption that the two estimators are independent the variance can be estimated as

$$ v\left({m}_t\right)=v\left(1-{p}_z\right)v\left({m}_{tnz}\right)+{\left(1- pz\right)}^2v\left({m}_{tnz}\right)+{m}_{tnz}^2v\left(1-{p}_z\right), $$

and, assuming simple random sampling and negligible finite population correction, *v*(1 − *p*_*z*_) = *p*_*z*_(1 − *p*_*z*_)/*n*.

The variance *v*(*m*_*tnz*_) is more difficult to estimate. Generally, the variance of the winsorised mean is used to estimate the variance of the trimmed mean because the two estimators are asymptotically equivalent. The winsorized mean is calculated by setting the extreme observations to the trimming quantiles *x*_*l*_ and *x*_*h*_. Suppose ${x}_i^{\prime },i\notin Z$ are the winsorized values and *n*_*t*_ is the sum of the weights in the non-trimmed part. Then the winsorised mean is

${m}_{wnz}={\sum}_{i\notin Z}{x}_i^{\prime }{w}_i/{\sum}_{i\notin Z}{w}_i$. Thus the winsorised mean is the weighted mean of the new values ${x}_i^{\prime },i\notin Z$. A variance estimator for the winsorised mean is

$$ v\left({m}_{tnz}\right)=v\left({m}_{wnz}\right)=\frac{1}{n_t\left({n}_t-1\right)}{\sum}_{i\notin Z}{w}_i{\left({x}_i^{\prime }-{m}_{wnz}\right)}^2. $$

Applying the formulas outlined above results in a trimmed, weighted mean and the corresponding variance estimator. For a discussion on the estimation of the variance of a trimmed mean, see also [12].

## Abbreviations

DRG: Diagnosis related groups
FOPH: Federal Office of Public Health
GP: General practitioner
KVG: Swiss Federal Health Insurance Act
KVV: Health Insurance Ordinance
PsyG: Federal Law on Psychological Professions

## Footnotes

1 An amortization rate of 20% implies that the computer is used for 5 years.

2 This roughly corresponds to the 1824 h that we assumed in Section Processing of survey results.
